# Advancing research for HIV prevention among African, Caribbean and Black men: Protocol for a multisite cross-sectional study in Ontario (weSpeak study)

**DOI:** 10.1097/MD.0000000000025662

**Published:** 2021-05-07

**Authors:** Winston Husbands, Josephine Etowa, Wesley Oakes, Francisca Omorodion, Isaac Luginaah, Egbe Etowa, Bishwajit Ghose, Josephine Pui-Hing Wong

**Affiliations:** a Ontario HIV Treatment Network, Toronto; b School of Nursing, Faculty of Health Sciences, University of Ottawa, Ottawa; c Department of Sociology, Anthropology & Criminology, University of Windsor, Windsor; d Department of Geography and Environment, Western University, London; e Daphne Cockwell School of Nursing, Faculty of Community Services, Ryerson University, Toronto, Ontario, Canada.

**Keywords:** African, Caribbean, and Black men, HIV, men's health, mixed method study, Ontario, weSpeak study

## Abstract

In Ontario, African, Caribbean, and Black (ACB) men account for almost 60% of the estimated number of HIV-positive people (through heterosexual contact), although they constitute less than 5% of the province's population. However, current HIV research, programming and policy in Ontario are not aligned with heterosexual ACB men's healthcare needs and interests, and fail to engage them in community responses to HIV.

weSpeak is a multisite (Ottawa, Toronto, London, and Windsor) project that is aims to:

1. assess the sociocultural and socio-political conditions that contribute to HIV related health disparities among ACB men,
2. examine social and behavioral vulnerabilities to HIV among ACB men, including their social identities related to race, class, gender and sexualities,
3. community engagement and mobilization part of the project, and
4. generate, appraise and share new knowledge, and support its translation into intervention and practice.

This will be a mixed method study comprising focus groups, in-depth interviews, and a survey to meet the data objectives. All data collection activities will take place at the same time in 4 cities in 3 sequential phases:

1. focus groups,
2. in-depth interviews, and
3. a questionnaire survey.

Service providers will participate in the concept mapping exercise to review the research findings and develop program, policy, and community-based initiatives to promote resilience and meaningfully engage heterosexual ACB men in community responses to HIV survey.

This study will provide evidence on:

1. heterosexual ACB men's experience of structural disadvantage and psychological factors is associated with HIV vulnerability.
2. heterosexual ACB men with greater internal resilience and social resources show greater risky behaviors, and
3. a conceptual Model of HIV vulnerability linking the potential internal and external factors that interact to influence HIV vulnerability.

This study will lead to better understanding of the structural determinants and the psychosocial risk factors of HIV transmission among ACB men in Ontario which will aid in designing evidence-based intervention programs, and thereby reduce their higher vulnerability to HIV and its associated consequences.

## 1 Introduction

Research evidence shows that heterosexual African, Caribbean, and Black (ACB) men experience social inequality that increases their vulnerabilities to HIV and other STIs.^[1,2]^ The causes behind this increased vulnerability are diverse and are generally linked to lack of health awareness, health literacy, engagement in risky behavior, systemic racism and inadequate capacity for availing preventive, and curative measures.^[3–7]^ In Canada, sexual health of ACB men are largely overlooked in the terms of research funding, prevention programs, and strengthening health services that can keep pace with the changing epidemiology of HIV. It is therefore likely that ACB men in Ontario face complex challenges that limit their access to and use HIV-related programs and services, including racist stereotypes that depict ACB men as wanton and sexually irresponsible.^[8–10]^ Men who are socially and economically marginalized may also find it ever harder to deal with their vulnerabilities and adopt sexual practices that reduce HIV transmission such as regular use of condoms and taking part in community programs and exchanges to highlight their special needs and challenges to the policy makers.

Previous studies have shown the importance of sociodemographic factors for proper utilization of condoms and relevant knowledge and attitude among general population.^[11–15]^ In contrast, not much research evidence has been generated on the health issues of ACB men, focusing on their individual and structural factors to reduce HIV-related risk and vulnerability, and promoting leadership and organizational capacity, and leadership to address the HIV/AIDS related disparities. To this regard, the proposed project was launched in late 2015 with the aim of reducing HIV vulnerabilities and promote resilience through active engagement of self-identified heterosexual ACB men in community HIV responses, programs, research, and policy. The specific objectives are to:

1. mobilize heterosexual ACB men to address sociocultural and socio-political conditions that contribute to HIV related health disparities;
2. examine social and behavioral vulnerabilities to HIV among ACB men, including their social identities related to race, class, gender, and sexualities;
3. identify the individual and structural factors that promote resilience and reduce HIV-related risk and vulnerability among heterosexual ACB men;
4. build individual, community, and organizational capacity and leadership to address HIV/AIDS disparities among heterosexual ACB men and ACB communities (including students, postdoctoral fellows, and engaged members within ACB communities) through research, programming, and policy; and
5. generate, appraise and share new knowledge, and support its translation into intervention, practice, and policy locally (in Ottawa, Toronto, London, and Windsor), provincially and nationally.

This is a 5-year research program and is designed to be integrative and sequential:

1. engagement and mobilization (years 1–5) to raise awareness among self-identified heterosexual ACB men about the social determinants of HIV vulnerabilities and set the stage for the research component;
2. innovative research (years 1–3) to investigate HIV vulnerabilities and resilience among heterosexual ACB men;
3. collective empowerment and capacity building (years 1–5) to build requisite skills, abilities and critical perspectives among heterosexual ACB men and stakeholders for effective HIV responses in ACB communities; and
4. KTE strategies (years 3–5) to mobilize heterosexual ACB men and stakeholders in the collaborative development and implementation of evidence-informed programming, research and policy to address HIV-related health disparities in ACB communities.

The overarching theme of the project is to examining the diverse social and psychological factors associated with vulnerability to HIV among self-identified heterosexual ACB men, including the impact of social identities related to race, class, gender, and sexualities; and identifying the individual and structural factors that promote resilience and reduce HIV and related health risks among self-identified heterosexual ACB men. Based on this, 3 core hypotheses will be tested: Hypothesis 1 (Risk Factors): Heterosexual ACB men experiencing greater structural disadvantage and/or having particular psychological factors will have greater HIV vulnerability. Hypothesis 2 (Protective Factors): Heterosexual ACB men with greater internal resilience and social resources will have less HIV risk related behaviors. Hypothesis 3 (Conceptual Model of HIV Vulnerability): Internal and external factors interact to influence HIV vulnerability.

## 2 Methods

### 2.1 Study design

This will be a cross-sectional mixed methods approach^[16]^ and will include multiple methods of recruitment, data collection and analysis, and KTE.^[17]^ Our research program will address the programming and policy gaps affecting heterosexual ACB men in Ontario, reduce HIV related and other social stigma, enhance HIV prevention messaging in ACB communities, and strengthen ACB men's involvement in community responses to HIV. The project was approved by the ethics review board of all affiliated universities. The project will be implemented in 3 overlapping phases:

#### 2.1.1 Phase 1: focus groups with ACB men and service providers (years 1–2)

In Phase 1 of our research component, our model for organizing the focus groups with self-identified heterosexual ACB men in Ottawa and Toronto is as follows:

1. people living with HIV/AIDS (PHAs): young men aged 16 to 24 and men aged 25+, French or English-speaking; and
2. non-PHAs: young men aged 16 to 24 and men aged 25+, French or English-speaking.

We anticipate 6 to 8 focus groups in Ottawa and a similar number in Toronto, with a maximum of 10 participants per group. In London and Windsor, we expect to organize 4 English language focus groups (PHAs: young men aged 16–24 and men aged 25+; non-PHAs: young men aged 16–24 and men aged 25+). The focus groups will discuss issues related to: participants’ understanding of HIV among ACB communities in Ontario, access to and availability of HIV and related health services; participants’ understanding of vulnerability, resilience, masculinity, heterosexuality, and health.

#### 2.1.2 Phase 2: in-depth interviews with ACB men (year 2)

We expect to recruit 25 men in Toronto, 15 in Ottawa, 10 each in London, and Windsor for individual interviews. We will use the same model outlined above for focus group to guide recruitment efforts. In addition, we will ensure equitable participation among men who self-identified as African, Caribbean, or Black. The in-depth interviews will draw from insights and critical issues that emerged from the focus groups. They also make it possible for participants to discuss issues related to vulnerability, relationships, sex and other sensitive, or personal issues that cannot be effectively broached in focus groups. The interviews and focus groups will also assess how vulnerability, resilience, heterosexuality, and masculinity emerge in their everyday experiences, and in relation to structural/systemic issues/themes such as race, class, and gender.

#### 2.1.3 Phase 3: survey of heterosexual ACB men (year 2–3)

In addition to focus groups and individual interviews, we will also use a self-administered survey to address 2 of our program objectives: examining social and psychological factors associated with vulnerability to HIV among self-identified heterosexual ACB men, including the impact of social identities related to race, class, gender, and sexualities; and identifying the individual and structural factors that promote resilience and reduce HIV and related health risks among self-identified heterosexual ACB men.

### 2.2 Analysis of qualitative data

Our data analysis will be guided by the population health promotion framework and critical social theories. All focus group and individual interviews will be audio-recorded and transcribed verbatim. We will use NVivo software for data management and to facilitate analysis. Our team will use thematic analysis. A theme is a pattern found in the information that describes and organizes the possible observations, or interpretations of phenomena identified in the data. This process will begin with the development of a coding framework informed by questions from the interview guides and a systematic approach that involves:

1. familiarizing with the data;
2. generating initial codes;
3. developing a coding tree to guide the coding of transcripts;
4. identifying themes;
5. reviewing, defining, and naming themes;
6. interpreting the narratives and stories; and
7. producing the report – a concise, coherent, logical, and non-repetitive account supported by vivid examples.

These iterative processes associated with qualitative analysis will ensure that preliminary interpretations are challenged and data are revisited in the light of further data collection and new insights into the data. During data analysis, preconceptions, and assumptions will be challenged, and consensus will be reached in understanding the data. Thematic analysis is suitable for this study because it is participatory and accessible; it enhances participation among team members (knowledge users, peer research associates, and academic researchers) in collaborative data analysis and interpretation. Themes derived from the qualitative analysis will also help us to inform the survey contents and interpret the results from our quantitative analysis of the survey data.

### 2.3 Sampling and recruitment

We estimate the survey sample size for each city based on the number of HIV tests among heterosexual ACB men over 5 years, 2007 to 2011, in relation to the total male ACB population 15 years and older in the respective cities from the 2011 National Household Survey, and a desired level of precision of 5%. For example, 15,465 tests were attributed to ACB men in Toronto, which represents 12% of Toronto's Black male population aged 15 and older in 2011. We will double the initial estimated sample size for Toronto. Moreover, based on the experience with the MaBwana Study, we will increase the sample size in all 4 cities by 25% to achieve greater statistical power and also make allowances for questionnaires/cases that are incomplete or ineligible. Our sample estimates are as follows: Toronto: revised sample size estimate (n = 324); recruitment target (n = 405) Ottawa: initial sample size estimate (n = 207); recruitment target (n = 259) London: initial sample size estimate (n = 160); recruitment target (n = 200) Windsor: initial sample size estimate (n = 140); recruitment target (n = 175). Based on the targets above, our sample for the survey will be close to 1039 self-identified heterosexual ACB men which, in the context of research with ACB communities in Canada constitutes a large sample.

### 2.4 Quantitative data analysis

#### 2.4.1 Descriptive and bivariate analyses

Descriptive statistics will be ascertained for the sample. Mean and median values will be generated for demographics (e.g., income) and sexual risk variables (age of sexual debut, lifetime number of sexual partners, number of partners in the past year), HIV vulnerabilities, and for all scales. Frequencies and percentages will be ascertained for relevant demographics (ethnicity, marital status, region of residence, employment status, educational attainment), key sources of sexual health information, key messages about sexuality, relevant sexual risk variables (condom use at last intercourse, frequency of safer sex with regular partners, frequency of safer sex with casual partners, ethnicity of sexual partner, gender of sexual partners), access to sexual health services (having a regular physician) HIV status, STI testing, and STI history. Bivariate analysis will be used to determine associations between key variables (demographics, psychological factors, and social/structural) and HIV vulnerability. Bivariate analysis will also be used to determine associations between key variables and sexual risks. Multivariate analysis Multivariate analysis will be conducted to determine the independent effect of each of the predictor variables such as socioeconomic disadvantage, discrimination, and masculine norms on vulnerability to HIV, controlling for theoretically relevant covariates.

The main course of the quantitative data analysis will be guided by our hypotheses and our program goal, that is, to investigate the relationships between HIV vulnerability and heterosexual ACB men's identities, resilience, social location, and social/structural contexts of their everyday life. Structural equation modelling techniques will be used to test the following hypotheses:

Hypothesis 1 (Risk Factors): Heterosexual ACB men experiencing greater structural disadvantage and/or having particular psychological factors will have greater HIV vulnerability.

1. Greater HIV vulnerability is associated with:
  a. greater everyday discrimination, and
  b. greater socioeconomic disadvantage (less income, less education).
2. Greater HIV vulnerability is associated with:
  c. more traditional masculine norms;
  d. negative attitudes towards condom; and
  e. lacking accurate HIV knowledge.

Hypothesis 2 (Protective Factors): Heterosexual ACB men with greater internal resilience and social resources will experience less HIV vulnerability.

Lower HIV vulnerability is associated with:

a. greater social capital; and
b. greater resilient coping styles.

Hypothesis 3 (Conceptual Model Exploration): Internal and external factors interact to influence HIV vulnerability.

1. The negative effect of masculinity on HIV vulnerability is mediated by condom use attitude
2. The negative effect of discrimination is moderated by protective factors (i.e., resilience and social capital).

### 2.5 Expected results

The anticipated outcomes of this program are:

1. Rigorous and culturally inclusive methodologies to engage ACB and other marginalized communities to generate evidence on the sources of vulnerabilities; best practices model to guide community HIV response; and theories on racialized heterosexuality and masculinities.
2. Strengthened research infrastructure and cultivation of the next generation of HIV researchers through training and mentorships of students and highly qualified personnel, and engagement of knowledge users in all phases of the program.
3. Communities of practice that support key stakeholders to access relevant information, synthesize evidence, build local and provincial networks, and leverage existing resources to set research priorities and initiate responsive research.
4. Increased awareness and understanding to enhance implementation of the provincial strategy on HIV, particularly the Strategy for ACB communities.

The team includes 30 members in 4 cities: 16 multidisciplinary academic researchers, 7 knowledge users (service providers, community-based researchers, policy makers, community advocates), and 7 collaborators who support the program of research and related activities by their varied but complementary skills and areas of expertise. Each local team will constitute a hub that is led by the local co-PI, and each hub will be responsible for the day-to-day implementation of project activities. Each hub will have its own local advisory committee (LAC), comprised of self-identified heterosexual ACB men and 1 member from the research team, to ensure that the interests of the primary constituents (ACB heterosexual men), local communities and community leaders are appropriately considered, and to advise the research team on all phases of the initiative. Various working groups comprised of members of the research team will drive the implementation of the initiative. These working groups will be organized around thematic and expertise areas. For example, there will be a qualitative data analysis workgroup that will lead the analysis of qualitative data and ensure quality control. The team structure, responsibilities and framework for collaboration of the various components and how individuals work together, will be set out in a term of reference to ensure a collegial, supportive, creative, and productive environment.

## 3 Discussion

The weSpeak study aims to explore the diverse social and psychological factors associated with vulnerability to HIV among self-identified heterosexual ACB men, including the impact of social identities related to race, class, gender and sexualities; and identifying the individual and structural factors that promote resilience and reduce HIV and related health risks among self-identified heterosexual ACB men. In brief, the project will use both quantitative and qualitative approaches to assess the experience of structural disadvantage and the psychological factors among ACB men and their link with HIV/AIDS vulnerability. Based on the existing literature and expert opinion, it is likely that heterosexual ACB men with greater internal resilience and social resources will have less HIV risk related behaviors, and that various proximal and distal factors (biological, social, cultural, environmental) interact to influence HIV vulnerability as well as HIV related health outcomes.^[18–21]^

A major strength of this project is its focus on addressing issues related to structural racial discrimination to build individual, community, and organizational capacity and leadership to address HIV/AIDS disparities among heterosexual ACB men and ACB communities. By doing so, the data and evidence can greatly enhance the translation of new knowledge in to programming and policy locally and in Ontario. This component will engage self-identified heterosexual ACB men, service providers, policy/decision makers to develop effective and empowering responses that address vulnerabilities (racism, social, and economic marginalization) and promote resilience and critical health literacy in the context of HIV and health. This process has been successfully used to develop a best practices model to address migration and HIV related issues affecting the mental health of PHAs. This component also promotes critical health literacy, which refers to “the degree to which people are able to access, understand, appraise, and communicate information to engage with the demands of different health contexts in order to promote and maintain good health across the life-course”.^[22–25]^ It will be implemented through an innovative use of concept mapping to simultaneously integrate knowledge translation and knowledge generation. For this purpose, 2 groups of knowledge users will be engaged: heterosexual ACB men, and service providers, policy/decision makers. Unlike end-of-project knowledge translation, this area of the research aims capture the insight and lived experiences of our participants to make sense of the research results from focus groups, in-depth interviews and the survey with a distinct purpose to generate evidence-informed solutions to address HIV vulnerabilities and promote resilience among heterosexual ACB men.

Lastly, the project will use the process of co-learning and co-creation of knowledge to inform the development of effective responses to HIV:

1. disrupt the conventional assumption/practice that only academic researchers hold the expertise to translate research results into knowledge to be consumed by knowledge users;
2. promote meaningful engagement of knowledge users in integrated KTE;
3. promote a sense of co-ownership and commitment that is critical for reducing the evidence-practice gaps; and
4. enable active participation of knowledge users to generate pragmatic, relevant, and inclusive strategies.

We will use concept mapping as an engagement tool and a research method. Concept mapping is an established method in program planning and evaluation research. It has recently been taken up in community-based research to explore health issues through the perspectives of affected individuals and communities. Concept mapping is a structured process in which participants’ input and ideas on a focused topic are captured and transformed into an interpretable visual representation (concept map).^[26–29]^ The concept map displays all of the ideas of the participants and shows how these ideas are related and the degree of relevance and importance.

There are many advantages in using concept mapping as a participatory approach to integrate, synthesize and translate research evidence into tangible HIV response strategies:

1. it facilitates equitable participation of all participants;
2. compared to conventional focus groups, the brainstorming process enables participants to generate a large number of specific ideas relevant to the chosen foci;
3. the resulting maps are expressed entirely in the language of the participants; and
4. it engages the participants in interpreting and finalizing the concept maps.

The expected the evidence will help strengthen multiple sector partnerships and collaboration in HIV responses of ACB communities; identifying initiatives to strengthen resilience; building critical health literacy; supporting ACB men's involvement in community responses to HIV; building capacity in CBR and policy analysis; and generating new knowledge to reduce HIV-related health disparities in ACB communities to address the HIV epidemic in Ontario and beyond. Although these issues sporadically appear in academic discourses and exchanges, there exists a dearth of concrete research evidence among ACB men in Ontario. From this perspective, findings from the current project will be a great resource for evidence-based policy making and healthcare promotion targeting the reduction of HIV vulnerability among ACB men in Canada.

## 4 Conclusion

The weSpeak project aims to build a supportive environment to meaningfully engage heterosexual ACB men and stakeholders to understand the contexts and conditions that shape health and wellbeing. Some of the notable outcomes of the proposed research program will include: strengthening multiple sector partnerships and collaboration in HIV responses of ACB communities; identifying initiatives to strengthen resilience; building critical health literacy; supporting ACB men's involvement in community responses to HIV; building capacity in CBR and policy analysis; and generating new knowledge to reduce HIV-related health disparities in ACB communities to address the HIV epidemic in Ontario and beyond. Critical health literacy will enable ACB men to realize self-determination and collaborate with communities, practitioners, and decision-makers to advance the Strategy on HIV for ACB Communities in Ontario. Through this program of research, our team will inspire researchers and communities to work together towards health equity. The proposed program of research, community engagement, capacity building, and knowledge translation and exchange (KTE) will meaningfully engage self-identified heterosexual African, Caribbean, and Black (ACB) men in critical dialogue about HIV and health, and strengthen community responses to HIV.

## Author contributions

**Conceptualization:** Winston Husbands, Josephine Etowa, Wesley Oakes, Francisca Omorodion, Isaac Luginaah, Josephine P Wong.

**Formal analysis:** Winston Husbands, Josephine Etowa, Wesley Oakes, Francisca Omorodion, Isaac Luginaah, Josephine P Wong.

**Funding acquisition:** Winston Husbands, Josephine Etowa, Wesley Oakes, Francisca Omorodion, Isaac Luginaah, Josephine P Wong.

**Investigation:** Winston Husbands, Josephine Etowa, Josephine P Wong, Wesley Oakes, Francisca Omorodion, Isaac Luginaah, Egbe Etowa.

**Methodology:** Winston Husbands, Josephine Etowa, Wesley Oakes, Josephine Wong, Francisca Omorodion, Isaac Luginaah, Egbe Etowa.

**Project administration:** Egbe Etowa, Bishwajit Ghose.

**Resources:** Isaac Luginaah.

**Visualization:** Bishwajit Ghose, Josephine Etowa.

**Writing – original draft:** Bishwajit Ghose.

**Writing – review & editing:** Bishwajit Ghose, Josephine P Wong, Winston Husbands, Josephine Etowa, Francisca Omorodion, Isaac Luginaah, Wesley Oakes, Ebbe Etowa.
